# Antimicrobial Silver Multilayer Coating for Prevention of Bacterial Colonization of Orthopedic Implants

**DOI:** 10.3390/ma13061415

**Published:** 2020-03-20

**Authors:** Martin Fabritius, Amir Andreas Al-Munajjed, Christiane Freytag, Henriette Jülke, Markus Zehe, Thomas Lemarchand, Jacobus J. Arts, Detlef Schumann, Volker Alt, Katrin Sternberg

**Affiliations:** 1 Aesculap AG, Research and Development, Am Aesculap-Platz, 78532 Tuttlingen, Germany; detlef.schumann@aesculap.de (D.S.); katrin.sternberg@aesculap.de (K.S.); 2 Bio-Gate AG, Neumeyerstraße 28-34, 90411 Nuremberg, Germany; amir.al-munajjed@bio-gate.de; 3 FREY_TOX GmbH, 04916 Herzberg, Germany; Christiane.Freytag@web.de (C.F.); FREY-TOX@t-online.de (H.J.); 4 QualityLabs, Neumeyerstr. 46a, 90411 Nuremberg, Germany; markus.zehe@qualitylabs-bt.de; 5 TPL Path Labs, Sasbacher Straße 10, 79111 Freiburg, Germany; lemarchand@tpl-path-labs.com; 6 Department of Orthopedic Surgery, Research School CAPHRI, Maastricht University Medical Centre, 6202 Maastricht, The Netherlands; j.arts@mumc.nl; 7 Department of Trauma Surgery, University of Regensburg, Franz-Josef-Strauss-Allee 11, 93053 Regensburg, Germany; volker.alt@ukr.de

**Keywords:** periprosthetic joint infections, infection prophylaxis, *Staphylococcus epidermidis*, in vivo osteomyelitis model

## Abstract

Due to increasing rates of periprosthetic joint infections (PJI), new approaches are needed to minimize the infection risk. The first goal of this study was to modify a well-established infection model to test surface-active antimicrobial systems. The second goal was to evaluate the antimicrobial activity of a silver multilayer (SML) coating. In vitro tests with SML items showed a >4 Log reduction in a proliferation assay and a 2.2 Log reduction in an agar immersion test (7 d). In the in vivo model blank and SML coated K-wires were seeded with ~2 × 10^4^ CFU of a methicillin-sensitive Staphylococcus epidermidis (MSSE) and inserted into the intramedullary tibial canal of rabbits. After 7 days, the animals were sacrificed and a clinical, microbiological and histological analysis was performed. Microbiology showed a 1.6 Log pathogen reduction on the surface of SML items (*p* = 0.022) and in loosely attached tissue (*p* = 0.012). In the SML group 7 of 12 SML items were completely free of pathogens (cure rate = 58%, *p* = 0.002), while only 1 of 12 blank items were free of pathogens (cure rate = 8%, *p* = 0.110). No silver was detected in the blood or urine of the SML treated animals and only scarcely in the liver or adjacent lymph nodes. In summary, an in vivo infection model to test implants with bacterial pre-incubation was established and the antimicrobial activity of the SML coating was successfully proven.

## 1. Introduction

Periprosthetic joints infection (PJI) is a severe complication for patients undergoing a joint replacement procedure that can lead to a straining month-long treatment process of early implant revision, multiple re-revisions or even an amputation of the infected limb. While infection rates after primary implantation are relatively low (0.5% to 2%) [1,2,3], they increase dramatically in the case of an implant revision (>10%) [4,5]. If the implant is revised due to a previous infection, the risk to develop a subsequent infection is even higher (~26%) [6]. Besides the high risk for the patient, PJI is also a tremendous economic burden to the healthcare system. Kasch et al. found that in Germany the direct hospital care costs for the management of a septic revision total knee arthroplasty (TKA) are about twice as high as for an aseptic failure [7]. Other papers conclude that the costs for a septic revision are 3 to 4 times that of an primary implantation [8,9]. 

Early (<3 month after surgery) or delayed infections (3–24 month after surgery) are caused by exogenic pathogens entering the surgical wound during surgery [4]. While there is a controversial discussion on which pathogens are the most relevant to cause PJI, it is generally agreed that Staphylococci species are predominant [4,10,11,12,13,14]. The main focus of scientific as well as public interest is given to *Staphylococcus aureus* (*S. aureus*) due to its high virulence and an increased awareness of antibiotic resistances that renders methicillin resistant *Staphylococcus aureus* (MRSA) the biggest threat to develop PJI. Nevertheless, there are various papers and clinical case studies that highlight the importance of coagulase-negative Staphylococci (Co-NS) e.g., *Staphylococcus epidermidis* (*S. epi*) to be the predominant pathogens causing PJI [4,11,12,13,14,15].

While early infections are mostly caused by very virulent pathogens like *S. aureus*, *Escherichia Coli or Pseudomonas aeruginosa*, delayed infection are caused by less virulent pathogens like Co-NS or Propionibacterium species [16,17,18,19]. Early infections are relatively easy to detect by swelling, increased temperature or pain. In contrast, delayed infections are mostly clinically unobtrusive, with delayed and/or nonspecific signs [17,19,20]. If an infection is detected in less than three weeks after implantation, there is a reasonable chance to treat it with the least invasive treatment option of debridement, antibiotic treatment and implant retention [17]. In the case of delayed or chronic infections, the implant most likely has to be revised. This means that delayed infections caused by Co-NS pathogens like a *Staphylococcus epidermidis* are even more dangerous and harder to treat than early infections caused by *S. aureus* [4,12,13,14,19,21].

Even though operations are carried out under strict hygiene measures, a perioperative contamination from the air or the patients skin can occur [22,23,24]. The immune system is well capable to address high loads of over 10^6^ pathogens. However, in the presence of a foreign body material like an implant, as little as 100 pathogens suffices to cause a severe infection [25]. This is caused by the biofilm formation of pathogens on the artificial surface, which renders the bacteria practically immune to host immune attacks or antibiotic treatment [22]. Therefore, it is of great interest to prevent the biofilm formation on the implants surface. To minimize the infection risk, the thorough implementation of prevention guidelines is essential [26]. In addition to that, technical solutions that protect the implant surface against bacterial colonization should be developed and transferred into clinical practice.

Many techniques and antimicrobial systems have been reported in literature, ranging from active antibiotic release devices to contact killing surfaces [27,28,29,30,31]. Silver is a long known antimicrobial substance, which is successfully applied in various clinically implemented and currently available implant systems on the market [32].

The MUTARS^®^ tumor prosthesis is galvanically silver coated (m(Ag) = 0.33–2.89 g) and is widely used in Europe, Australia and various Asian countries [33,34]. Hardes et al. Donati et al. and Zajonz et al. reported a successful treatment and a reduced infection rate with the silver coated MUTARS^®^ prosthesis compared to a standard implant [35,36,37,38]. The Agluna^®^ technology dopes a titanium surfaces with silver ions by an electrochemical process [39]. In a case control study with 170 patients, Wafa et al. reported lower rates of early PJI when Agluna^®^-treated tumor implants were used [15]. The PorAg^®^ coating is a dual layer system with a silver base layer (1 µm thick) and a rigid top layer of TiAg20N (0.1 µm thick) [40]. Scoccianti et al. reported the successful use of PorAg^®^ coated tumor prosthesis in 33 patients without negative side-effects like argyria [41]. The AgPROTEX^®^ coating is a Hydroxyapatite/Ag_2_O system which is applied to metal surfaces by flame spraying (T = 2700 °C) [42]. The coating is approved for the use in primary total hip arthroplasty (THA) in Japan [43]. According to Eto et al. no adverse reactions were detected in the first clinical application of AgPROTEX^®^ coated primary THA implants (m(Ag) = 1.9 to 2.9 mg) with 20 patients [44]. However, all these silver-based systems are only suitable for metal implants and cannot be applied to polymer surfaces. This leaves the Polyethylene (PE) liner unprotected, even though it is known that the PE components are most often affected by PJI and carry the highest bacterial load [45].

The silver multilayer coating (SML) (HyProtect^TM^, Bio-Gate, Nuernberg, Germany) can be applied to both metal and polymer components. Silver clusters are embedded in a polysiloxane (SiOxCy) matrix and act as a reservoir for the release of silver ions that are anti-microbially active on the coating surface. Therefore, elementary silver itself is not in direct contact with the surrounding bone or tissue. Due to its ultra-thin layer (~90 nm), SML maintains the porosity of nano-structured/porous surfaces intact and does not seal them, which is important for osseointegration. The combination of osteoconductive and osteoinductive biomaterials like calcium phosphate, hydroxyapatite, bisphosphonate and silicates in combination with nanoscale therapeutics like BMP-2 have also shown to support bone regeneration, which proves beneficial for secondary implant stability [46,47].

A paper by Khalilpour et al. reported on various successful tests of the SML coating like the in vitro antimicrobial activity, no cytotoxicity according to ISO 10993-5 and ex vivo antimicrobial activity [48]. In a recent case study, the SML coating was used in a successful knee arthrodesis after recurrent periprosthetic knee infection, and silver levels in the drainage fluid and blood were evaluated. Silver blood concentrations after 48 h remained under the detection limit of 2 ppb, whereas the silver concentrations in the wound drainage fluid reached 170 and 57 ppb 24 and 48 h post-operatively, respectively [49].

In most established in vivo osteomyelitis models, an implant is placed in the tibia medullary canal, and a bacteria suspension is injected afterwards [27,28,42,50,51,52]. This leads to a localized high concentration of pathogens with no uniform distribution along the tibia canal. In case of drug release systems, this is of minor importance as the released drug is able to target present pathogens in a larger vicinity of the implant. In contrast, surface-active coatings are unable to target these pathogens, which can subsequently grow in the more distant tissues. Once an infection is established, high numbers of pathogens are released into the surrounding tissues, which leads to an overpowering of the surface-active system, and therefore, no antimicrobial activity can be proven with such models. 

The objective of this study was (i) to establish a suitable in vivo osteomyelitis model in rabbits and (ii) to evaluate the antimicrobial activity of a silver multilayer coating (SML) under realistic pre-clinical conditions. We hypothesized that the SML coating can significantly reduce the CFU count on the K-wire surface at explantation by a minimum of 1 Log reduction compared to the initial CFU count.

## 2. Materials and Methods

### 2.1. Implant Items

**Figure 1:**
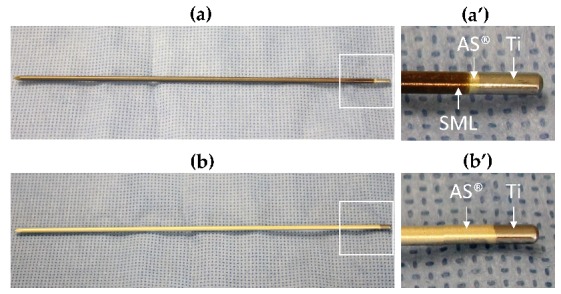
(**a**) Test item: SML-coated AS^®^/titanium K-wire, (**a’**) shows the two coatings in magnification. SML = bronze, AS^®^ = golden. (**b**) Blank item: AS^®^-coated titanium K-wire, (**b’**) shows the AS^®^ coating in magnification. The silver color at the blunt end of each K-wire shows the uncoated titanium surface.

Gamma-sterilized pure titanium K-wires with a diameter of 2.0 mm (MEDE Technik GmbH, Emmingen-Liptingen, Germany) and a length of 150 mm were used for the study. To provide an inert surface, all K-wires were coated with the Advanced Surface^®^ ceramic multilayer coating (AS^®^, Aesculap AG, Tuttlingen, Germany) over a length of ~140 mm. [53,54] The test items were coated with the SML coating by Bio-Gate AG (Nuremberg, Germany) (Figure 1).

#### SML Coating

Test items were coated with the SML coating in a three-step process. In a first step, a SiO_x_C_y_ base layer was deposited on the respective surface by chemical vapor deposition (CVD). In a second step, silver clusters (~2.7 µg/cm^2^) were deposited on the base layer by physical vapor deposition (PVD), and in a third step covered with a SiO_x_C_y_ top layer. This resulted in a coating with a thickness of ~90 nm. The coated items were packed individually, and gamma sterilized (BBF Sterilisationsservice GmbH, Kernen-Rommelshausen, Germany). Previous publications give more detail on the SML coating [48,49].

Every SML coating batch was characterized after production by various test methods on planar surface aluminum coated PET foil (dummy items). The chemical structure of the SML coating was analyzed by FTIR spectrometry (Tensor 27; Bruker Optik GmbH, Ettlingen, Germany). The silver content was determined by inductive coupled plasma-optical emission spectroscopy (ICP-OES) according to EN ISO 11885 (Seibersdorf Labor GmbH, Seibersdorf, Austria). The coating thickness was measured by spectral ellipsometry (IFAM Fraunhofer Institut, Bremen, Germany). Each of these tests were performed in triplicate on non-sterilized dummy items. Additionally, titanium test plates were coated simultaneously and analyzed according to ISO 10993-5 to prove non-cytotoxic behavior.

### 2.2. In Vitro Antimicrobial Activity

Prior to the in vivo tests various in vitro tests were performed to re-verify the antimicrobial activity of the SML coating on surfaces relevant to orthopedic implants.

#### 2.2.1. QualiScreen^®^ Tests

The in vitro antimicrobial activity of blank and test items was evaluated using a proliferation assay described previously [55,56]. In brief, four replicates of SML coated test items and 4 blank items were incubated in 10% human plasma for 1 h and subsequently washed in 1 × phosphate buffered saline (PBS) for 10 min. Afterwards, the items were incubated in a cell suspension of 5 × 10^6^ culture forming units (CFU)/mL MSSE (RKI 10-00621) at 37 °C for 1 h to allow bacterial cells to adhere to the item surface. Loosely attached bacteria were then removed by rinsing. Subsequently, the remaining cells were incubated for 18 h at 37 °C (challenge time). After removal of the test items, 50 µL of tryptic soy broth (TPS) was added to each well. The bacterial growth of the remaining daughter cells was monitored with a microplate reader over a period of 48 h.

#### 2.2.2. Agar Immersion Test

To mimic the in vivo situation, we subsequently tested the antimicrobial activity in an agar immersion test. SML coated items and blank items (AS^®^ coated titanium) were incubated with MSSE (RKI 10-00621) as described in Section 2.3. The seeded K-wires were immersed in a pre-prepared agar slurry (1% Agar and 0.1% TSB) and incubated for 24 and 72 h as well as 7 days. Afterwards, the K-wires were sonicated (3 min) and vortexed (30 s) in PBS to detach adherent bacteria from the item surface. The number of colonies was determined by agar plate count. Each measurement was performed in triplicate.

### 2.3. In Vivo Study Design

To determine the antimicrobial activity of the SML an existing in vivo model published by Alt et al. was adapted and an MSSE was used as contaminant [28]. The modification of this model consisted of the us of pre-incubated implants with bacteria in order to assess the effect of the silver coating on the implant surface compared to the inoculation of bacteria into the intramedullary canal after implantation of the K-wire in the referenced model. SML coated and blank K-wires were used as implants. Both implants were loaded with MSSE before implantation (see below). The studies were approved by the German regional authority of Brandenburg (2347-A-4-10-2014) in compliance with the EU principles for animal care.

#### 2.3.1. Pilot Studies

In order to establish and validate the in vivo model, two pilot studies with 6 SPF New Zealand White Rabbits (Envigo, 5800 Venray, The Netherlands) each with a body weight from 3.6 to 4.0 kg where performed. Three animals were treated with a blank K-wire and three animals with an SML coated K-wire. The operation procedure, microbial contamination and subsequent analysis were identical with the one of the main study described below. The silver analysis in organs, blood and urine was only performed in the main study.

#### 2.3.2. Main Study

The study included 27 SPF New Zealand White Rabbits (Envigo, 5800 Venray, The Netherlands) with a body weight from 3.7 to 4.4 kg. As the SML is only surface-active and has no widespread release of antimicrobial substance, we seeded the implants directly with pathogens, instead of injecting bacteria inoculum into the tibia canal. With this approach, we were able to guarantee a contact of pathogens with SML and simultaneously avoid an uncontrolled distribution of bacteria in the tibia canal, which might lead to false negative results.

The K-wires were contaminated in vitro with ~2 × 10^4^ colony-forming units (CFUs) of MSSE RKI 10-00621 and implanted into the intramedullary canal of the tibia in the rabbit. The method to determine the bacterial load on the K-wire surface is described in Section 2.4.2.

The test group of twelve animals received a SML coated K-wire (test item), while the control group received blank K-wires (blank item). Both were contaminated with identical loads of bacteria. The remaining three animals were implanted with test items without any microbial load. These three animals served as control to differentiate between implantation process-related and bacterial contamination-related lesions in the histological examination.

### 2.4. Bacteria

#### 2.4.1. Bacterial Strains

MSSE RKI 10-00621 was used as a contaminant in this study. RKI 10-00621 is a clinical isolate from a patient with PJI. It was obtained from the national reference center for Staphylococci and Enterococci of the Robert Koch Institute (RKI) in Wernigerode, Germany.

#### 2.4.2. Bacteria Cultivation and Pre-Incubation of Implants

The bacterium was grown in 20% Tryptic soy broth (TSB) for 4 h at 37 °C. Prior to implantation, two SML K-wires (per test tube) were contaminated in a test tube over a length of 9 cm by incubation in a bacterial solution of ~1 × 10^6^ CFU/mL for 30 to 60 min under dynamic conditions. In pretests, it was proven that the bacterial load level on the item surface can be preserved for this time period. The bacteria were freshly prepared and in logarithmic phase for the controlled contamination of the implant. No biofilm was present at the time of surgery. Subsequently to incubation, non-adherent bacteria were removed by rinsing the test item in PBS for 10 min. One K-wire was implanted into the tibia canal, and the other K-wire was used to determine the pathogen load on the surface at the time of implantation. The pathogens were removed by sonication and vortexing, and the bacterial count was determined by agar plate count.

### 2.5. Surgery

Intramuscular anesthesia was performed using ketamine (40 mg kg^−1^ body weight) and xylazine (6 mg kg^−1^ body weight). Perioperative analgesia was applied subcutaneously (Butorphanol, 0.5 mg kg^−1^ body weight).

**Figure 2:**
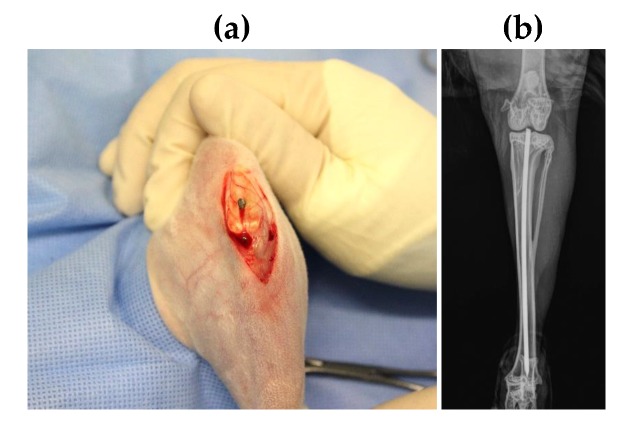
(**a**) Image of SML coated K-wire implanted in intramedullary canal, (**b**) Post-operative X-ray image of the rabbit tibia with the K-wire placement.

Surgery was carried out under aseptic conditions according to the model published by Alt et al. and no systemic antibiotics were given [28]. The left hind leg was shaven, fixated, disinfected and draped in sterile covering. The tibia was approached via an infrapatellar skin incision and subsequent preparation of Ligamentum patellae and Tuberositas tibiae. The Tuberositas tibiae was sharply penetrated and the medullary channel opened with a 2.0 mm K-wire. A template K-wire was inserted along the medial cortical bone until the proximal part of Malleolus medialis for channel preparation. After removal of the template, one implant per animal was applied. The implant was inserted into the prefabricated channel at a length of approx. 10 cm and fixated in the proximal part of the Malleolus medialis. The K-wires were contaminated over a length of 9 cm to make sure that no pathogens enter the knee joint. Table 1 lists the group arrangement. Group 1 (test item) and Group 2 (blank item) were seeded with 2 × 10^4^ CFU MSSE. Group 3 (test item) served as a negative control for histology to determine the lesions induced by the implantation process. The protruding part of the implant was removed, and the implant site was rinsed with Octenisept^®^ and NaCl 0.9%. Afterwards the wound was closed, and post-operative X-ray control was performed (Figure 2).

After 7 days post operation, the animals were put under general anesthesia (ketamine 40 mg kg^−1^ body weight, xylazine 6 mg kg^−1^ body weight) prior to euthanasia (T61 intravenously). The implantation site and the surrounding tissue were examined macroscopically, and samples were harvested. For microbiological examination, the implant and bone marrow were collected. The proximal tibia was transversally cut open for implant removal. Bone marrow was extracted from the central tibial bone using sterile instruments. It was manually mixed and halved for microbiological analysis and silver level measurements. For histological evaluation, the tibial bone was segmented into three parts by two transversal cuts. The proximal cut was set distally to the Tuberositas tibiae, and the distal cut was placed proximal to the Malleoli (Figure S1). Both proximal and distal segments were immersed in neutral buffered formalin for subsequent histopathological examination.

### 2.6. Sample Collection for Silver Measurement

For pharmacokinetic examinations, samples of whole blood and urine were collected from groups 1 and 2 at defined time points. Samples for determination of zero levels were gathered 7 days prior to surgery at maximum. Subsequently, samples were taken on days 1 and 7 post operation. Approximately 1.5 mL whole blood was taken using a lithium-heparin-tube. Urine samples were harvested as 24-h-samples prior to surgery and on day 1 post operation, whereas on day 7 post operation, urine was favorable collected as punctate. For extended determination of silver levels, situs associated lymph nodes (Lnn. poplitei and Lnn. inguinales), and liver were collected.

### 2.7. Evaluation Methods

#### 2.7.1. Clinical Assessment for Infection

Before harvesting of the bone, the knee joint and the surrounding of the implantation site were evaluated for clinical signs of inflammation or swelling. Therefore, areas surrounding the insertion site being directly net to the knee joint, adjacent soft tissue as well as the external structure of the tibia were examined regarding swelling, edema, excessive fluids and pus.

After implant removal and transversal opening of the tibia, the bone marrow was assessed for signs of infection. The quality of the bone marrow was defined as follows. Physiological quality: grey-white–pinkish–light red coloration, not washed-out, no watery phase, formed structure. Inflamed quality: reddish–red–bright red–dark red coloration, washed-out, with aqueous phase and unformed structure. In case of multiple findings, the most severe grade was taken into account for evaluation. In order to standardize qualitative description, macroscopic evaluation was performed in a blinded manner by the same investigator in all cases.

#### 2.7.2. Microbiological Assessment for Infection

At explantation, it was noted that varying amounts of bone marrow or tissue adhered to the K-wires. To avoid a distortion of the CFU count, the implants were gently rinsed in phosphate buffered saline (PBS) to remove the attached tissue (rinsing solution = “rinsing sol.”) Subsequently the K-wires were sonicated (3 min) and vortexed (30 s) in PBS to detach adherent bacteria from the item surface (“K-wire”). The bone marrow was collected as described in Section 2.5 and was sonicated in PBS to harvest containing pathogens. Bacterial contamination in the bone marrow was standardized to 1 g (“BMstd”). The three bacterial suspensions (K-wire, rinsing sol., BMstd) were diluted (1:1, 1:10), plated out on agar plates (1000 µL) and incubated over night at 37 °C. The number of colonies was determined by visual agar plate count. 

#### 2.7.3. Histological Evaluation

Tibia parts (proximal + distal) for histological examination were fixed in 4% phosphate-buffered formaldehyde solution for 72 h. The formalin-fixed bones were cut with a band saw sagittally, para-sagittally and longitudinally into a total of five ~3 mm slabs, according to Figure S1, 1 transverse slab and 2 longitudinal slabs for the proximal segment and 2 longitudinal slabs for the distal segment. The slabs were then demineralized for 1 to max 2 weeks in a solution containing formic acid (5% formic acid in distilled water) and dehydrated and embedded in paraffin. Formalin-fixed paraffin-embedded tissues were cut into 3 µm thick tissue sections and stained with haematoxylin and eosin (HE) or Gram stain according to a method derived from Brown and Brenn [57]. In order to maximize the chances to detect bacterial colonies and inflammatory lesions, step sections at least 50 µm apart were prepared from each block. The presence or absence and if present, an evaluation of the extent of osteomyelitis and bacterial colonies at the site of the contaminated K-wire insertion was carried out at the proximal and distal ends of the tibia. A qualitative ordinal scoring approach (ordinal non-continuous categorical response variables) (usually improperly referred to as “semi-quantitative scoring”) was used for the histological evaluation following current animal disease model literature and pharmaceutical development pathological safety/efficacy investigations, as well as Annex E of DIN EN ISO 10993-6:2014-12 [58,59]. It was based on the presence or absence of a microscopic change, finding or lesion and a scoring of extent and magnitude using a relative or absolute scale in a 6-category system: none, minimal, slight (mild), moderate, marked and very marked (severe). No morphological change quantification with digital section and image analysis was carried out, as most often the change was no longer present in a majority of sections.

#### 2.7.4. Silver Levels

For silver level analysis, samples collected according to Section 2.6 were used. The urine samples were centrifuged, and an aliquot of each sample was diluted and measured with inductively coupled plasma mass spectrometry (ICP-MS) according to ÖNORM EN ISO 17294-2 [60]. Blood, liver and lymph nodes were digested in an UV-digestion apparatus by using nitric acid and hydrogen peroxide and analyzed as described above. The detection limit of the ICP-MS depends on the sample quantity, which is analyzed. The following detection limits apply: blood (d0, d1, d7) < 6 µg/kg; urine (d0, d1, d7) < 0.6 µg/kg; liver < 3–6 µg/kg. Due to the varying sample quantity, the detection limit for the lymph nodes fluctuated considerably.

#### 2.7.5. Statistical Analysis

Statistical analysis was performed with Minitab R 17 (Minitab LLC, PA, USA). Due to a significant deviation from a normal distribution of the SML group, a Mann–Whitney test was used to evaluate whether there is a difference between the contamination at the beginning (implantation) and the end (explantation) of the experiment. This was done for the three respective bacterial suspensions (K-wire, rinsing sol., BMstd). We also compared the CFU count of SML coated and blank items after explantation. A Mann–Whitney test was used to evaluate whether there is a difference between the bacterial counts in the bacterial suspensions K-wire and rinsing sol. Differences were considered as significant for *p* < 0.05.

## 3. Results

### 3.1. In Vitro Antimicrobial Activity

**Figure 3:**
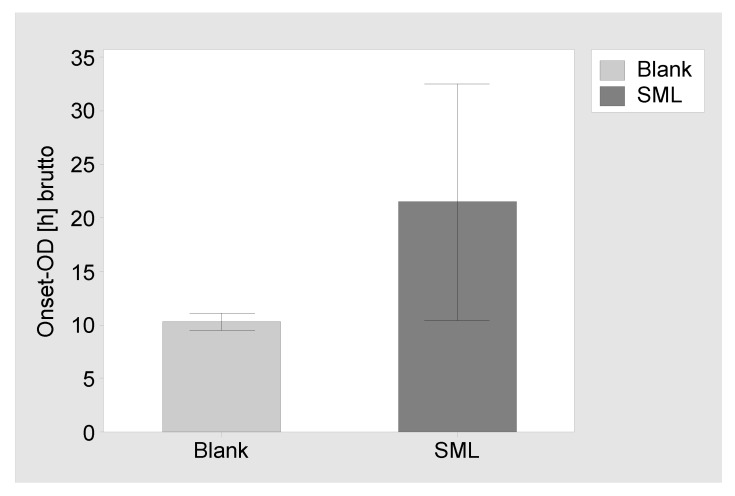
Results of the proliferation assay. Brutto Onset-OD time and 95% confidence interval for blank items and SML coated items. The netto Onset-OD of 11.2 h relates to a >4 Log reduction.

Proliferation assay: The SML coated items and blank items were tested against MSSE (RKI 10-00621). The blank items showed a brutto Onset-OD of 10.3 ± 0.8 h, while the SML items showed a brutto Onset-OD of 21.5 ± 11.0 h. This results in an average netto Onset-OD of 11.2 ± 7.3 h, which relates to a >4 Log reduction [55,56] (Figure 3).

**Figure 4:**
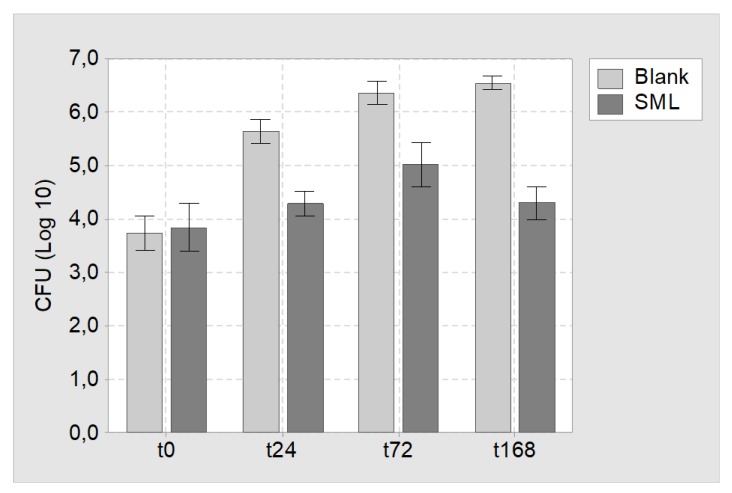
Results of the agar immersion test. Mean CFU count and 95% confidence interval for blank and SML coated K-wires at time point t = 0 h (t0), t = 24 h (t24), t = 72 h (t72) and t = 168 h (t168).

Agar immersion test: SML and blank items were challenged with MSSE (RKI 10-00621) and incubated for 24, 72 and 168 h in agar slurry. At the respective times the SML coated items showed a CFU reduction of 1.4 ± 0.2, 1.3 ± 0.3 and 2.2 ± 0.2 Log compared to uncoated blank items (Figure 4).

### 3.2. In Vivo Experiments

#### 3.2.1. Clinical Assessment

**Figure 5:**
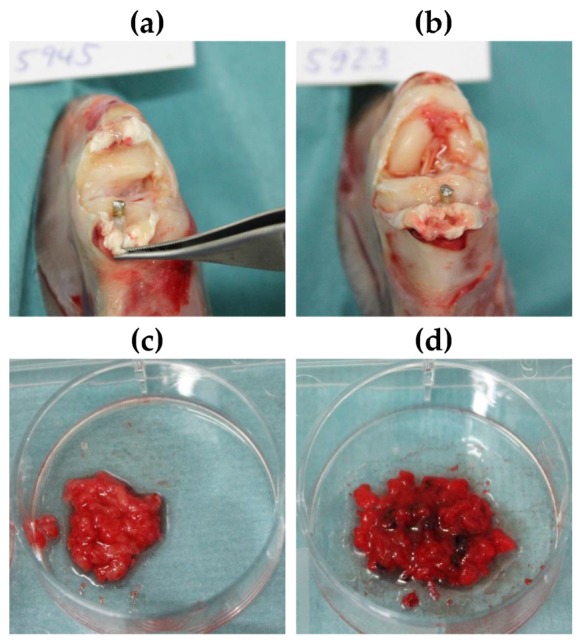
(**a**,**b**) Exemplary images of the knee joint postmortem of animals treated with (**a**) SML item and (**b**) blank item. (**c**,**d**) Exemplary images of bone marrow after explantation of (**c**) SML item and (**d**) blank item. (**c**) Physiological bone marrow was found in 11 of 12 animals. This equals a cure rate = 8%. (**d**) Fragmented and hemorrhagic aspects indicating osteomyelitis were found in 4 of 12 animals. This equals to a cure rate of 67%.

In general, the established infection model was considered to be mild, as septic arthritis of the knee joint was not detected by clinical observation in any case (Figure 5a,b). In 4 of 12 animals of the SML group signs of osteomyelitis were found in the bone marrow. This was marked by an increased red and/or washed-out coloration, as well as an unformed structure of the bone marrow and partial presence of an aqueous phase. In the other 8 cases of the SML group, the bone marrow was evaluated as physiological or “cured” (SML–cured 8/12 = 67%). On the contrary, bone marrow of animals from the blank group showed signs of osteomyelitis in 11 of 12 cases and only one animal was documented as physiologic or cured (Blank–cured 1/12 = 8%) (Figure 5).

#### 3.2.2. Microbiological Assessment for Infection

**Figure 6:**
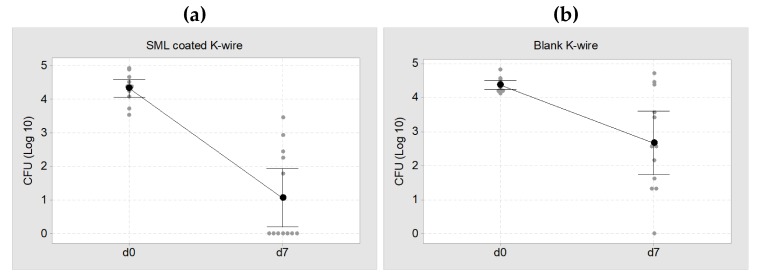
Individual value plot of CFU at d0 (implantation, ~2 × 10^4^ CFU) and d7 (explantation). (**a**) SML coated K-wires, mean CFU = 353 ± 529 CFU. This equals a cure rate of 58% (**b**) Blank K-wires, mean CFU = 9.282 ± 10.585 CFU. This equals a cure rate of 8%. The black dots represent the mean value of each group with a 95% confidence interval.

In the microbiological evaluation, 7 of 12 SML coated K-wires were free of pathogens and the remaining 5 K-wires showed a distinct CFU reduction compared to implantation. This equals a cure rate of 58% (*p* = 0.002). The mean pathogen count for the whole 12 SML K-wires was 353 ± 529 CFU. In the control group, only 1 of 12 K-wires was free of pathogens and the CFU count on the whole twelve blank K-wires was 9.282 ± 10.585 CFU (Figure 6). This equals a cure rate of 8% (*p* = 0.110).

The results of the Mann–Whitney test for the three bacterial suspensions (K-wire, rinsing sol., BMstd) are listed in Table 2. A comparison of the CFU count at implantation and explantation showed a decrease in all bacterial suspensions and the reduction is significant for both blank and SML coated items (*p* < 0.05, Table 2). The CFU reduction was always higher for SML coated K-wires than for blank K-wires.

**Figure 7:**
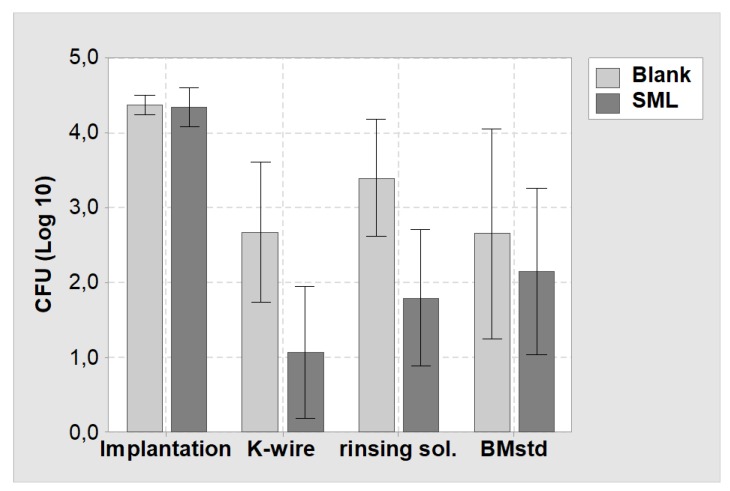
Mean values and 95% confidence interval of the CFU count of the in vivo study. (Implantation) = CFU count at implantation on the K-wire surface. (K-wire) = Bacterial suspension derived from the K-wire surface at explantation. (rinsing sol.) = Bacterial suspension derived from rising the K-wire surface to remove attached tissue. (BMstd) = Bacterial suspension derived from the bone marrow (normalized to 1 g). Light grey = Blank items, dark grey = SML items.

Comparing the CFU count at explantation for SML and blank group showed a 1.6 Log reduction for the SML coated items on the K-wire surface (*p* = 0.022) and in the rinsing solution (*p* = 0.012, Table 3). The bone marrow of the SML group also exhibited less pathogens than the blank group (0.5 Log), yet the effect was less pronounced (Figure 7).

#### 3.2.3. Histological Evaluation

**Figure 8:**
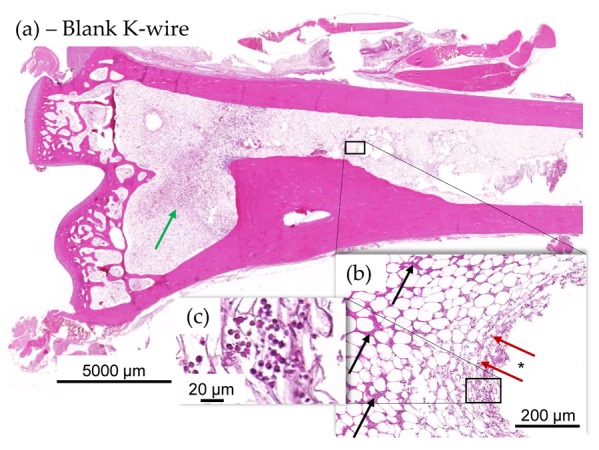
(**a**–**c**) Histology images of distal tibia of blank item animal. (**a**) There was a higher incidence of mild focal ongoing osteomyelitis in the distal tibia of blank K-wire implanted animals (**b**). Green arrows indicate new fibrous tissue (fibroplasia). Osteomyelitis was focal and showed evidence of several days old pus (black arrows) at periphery of implant, in the bone marrow and more recent exudate of intact and degenerated heterophils (red arrows and (**c**)) in the bone marrow immediately adjacent to the implant imprint (*), indicating active suppurative inflammation.

**Figure 9:**
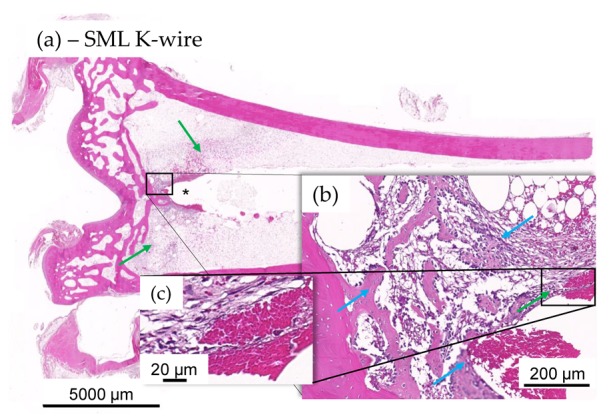
(**a**–**c**) Histology images of distal tibia of test item animal. No evidence of mild ongoing osteomyelitis along the K-wire in most SML K-wire implanted animal and stabilization was often seen to be more significant in the test item at tip of the K-wire imprint (*). Integration was by means of fibroplasia (green arrows) and new fibrous bone formation at host-implant interface ((**b**), blue arrows), devoid of any heterophilic infiltration (**c**).

**Figure 10:**
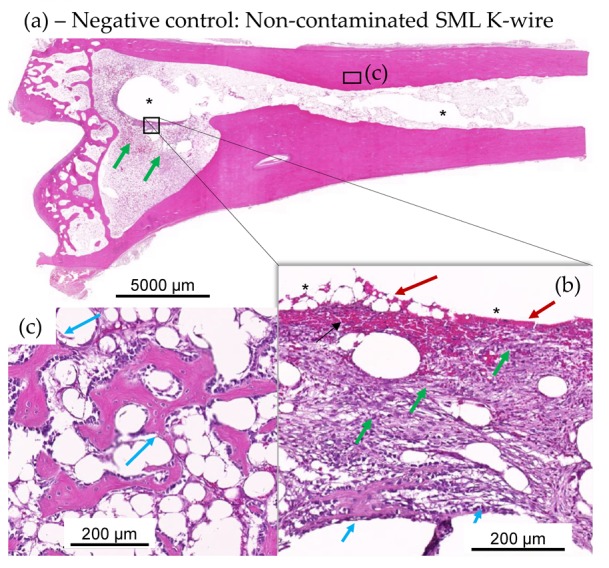
(**a**–**c**): Test item SML K-wire-implanted rabbits with no bacterial contamination, showing lack of suppuration and excellent implant stabilization by means of fibrous connective tissue (green arrows inset (**b**)) and recent new bone formation (see magenta arrows in insets (**b**,**c**)). Very recent new fibrous bone formation at host-implant interface (see short magenta arrows in inset (**b**)), devoid of any heterophilic infiltration, and more mature and older new fibrous bone (see longer magenta arrows in panel (**c**) from adjacent step section in metaphysis region adjacent the epiphysis EP). At the interface of the removed K-wire and the new connective tissue, there is limited red blood cell extravasation or hemorrhage (red arrows in inset (**b**)).

Histology showed less heterophilic infiltration/pus and fibroplasia in the distal tibia of animals with SML items, compared to the distal tibia of animals with blank items (Figure 8 and Figure 9). Similar degrees of inflammation and associated repair were noted in the proximal tibia of SML group and blank group animals. In general, the induced inflammation was very mild and barely above the one induced by the surgery alone. Very importantly, the new bone formation around the implant was very active in this disease model and comparable for test item and blank item. The negative control group (SML item with no bacterial contamination) showed no suppuration and excellent implant stabilization by means of fibrous connective tissue and recent new bone formation (Figure 10).

#### 3.2.4. Blood and Urine Analysis

**Figure 11:**
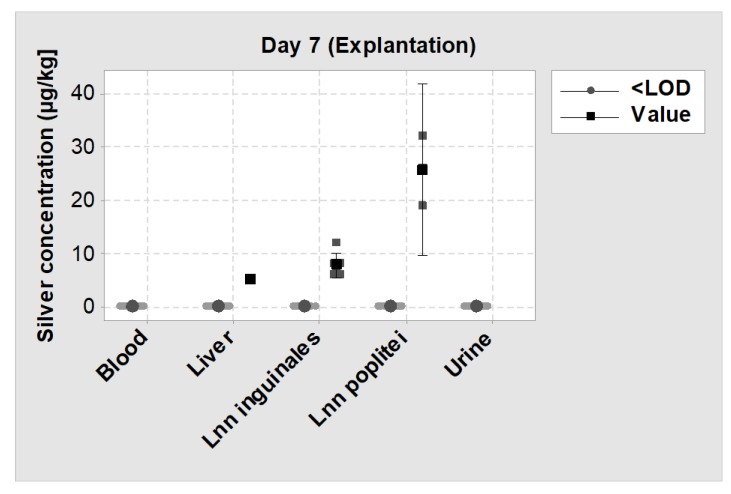
Individual value plot of silver levels measured by inductively coupled plasma mass spectrometry (ICP-MS) on day 7 (explantation) in blood, urine, liver and two lymph nodes. <LOD = Silver concentration was below the detection limit; Value = Silver concentration could be measured. The big symbols represent the mean value of each group with a 95% confidence interval.

No silver was detected in the blood and urine of all SML group animals at days 0, 1 and 7. In 1 of 12 animals, silver was detected in the liver (5 µg/kg), while in 11 of 12 animals, silver levels were below the detection limit. In Lnn poplitei, silver was detectable in 3 of 12 animals and in Lnn inguinales in 6 of 12 animals while in all other cases silver levels were below the detection limit (Figure 11).

## 4. Discussion

In the development of PJI, the initial step of bacteria adhering to the implant surface is of utmost importance. It initiates the cascade of bacterial proliferation and subsequent biofilm formation, which protects the bacteria from the host defense system. This contributes to inferior results in treatment options, as the susceptibility to antibiotics is dramatically decreased at this location. Therefore, the protection of the implant surface from bacterial colonization is potentially the most important step to prevent PJI.

The objective of this study was (i) to establish a suitable in vivo osteomyelitis model with pre-incubated implants with *Staphyloccocus epidermidis* in rabbits and (ii) to evaluate the antimicrobial activity of a silver multilayer coating (SML) under realistic pre-clinical conditions. Our study has some limitations that need to be taken into account when interpreting the results. Hischebeth et al. showed that MRSE infections are more difficult to cure than MRSA infections [21]. However, in our study, we used MSSE instead of MRSE due to safety aspects and to avoid unnecessary use of MRSE. Aspects such as antibiotic resistance, no differences in several bacterial properties such as proliferation, biofilm development and adherence are to be expected between MSSE and MRSE. The limitation of a relatively short test period of 7 days when compared to the timeframe of delayed infections (3–10 weeks) was accepted mainly for animal-welfare reasons. The test period was sufficient to identify the microbiological significant difference in the infection course. To test for complete clearance of the infection, further research with a longer test interval could be performed.

In literature, various models have been established to determine the antimicrobial activity of antimicrobial coatings most of which use MRSA as contaminant [52]. Recent findings emphasize that in clinical reality its “little brother” methicillin-resistant *Staphyloccocus epidermidis* (MRSE) is even harder to cure and spreads around the globe undetected [21,61]. In our study design, we therefore tested the antimicrobial activity of the SML coating against MSSE, which is associated with a strong biofilm forming capacity [62].

Most of the mentioned animal models are designed to test active release systems that address pathogens in the larger tissue region around the coated implant and therefore could actively treat an infection [27,28,50,51]. For example, Suhardi et al. reported the successful treatment of a PJI with an antibiotic releasing PE [27]. However, this approach has a major drawback for cementless endoprosthesis as they have the necessity of bone ingrowth which is essential to guaranty secondary implant stability. Antimicrobial substances like antibiotics or silver can have a cytotoxic effect on osteoblasts and thus may impair bone ingrowth around the implant [63,64]. Therefore, it is important to minimize the exposure to these substances while still guaranteeing an antimicrobial activity. Our measurements showed no silver in blood and urine and very low silver concentrations in the liver and adjacent lymph nodes, which proves a very limited systemic silver exposure. These findings are in line with the histopathological evaluation, which found very active new bone formation for both test item and blank item.

Another relevant aspect to take into account is the clinical approach currently used to treat PJI. The surgical techniques involve the thorough debridement of infected tissue, systemic antibiotic therapy and wound irrigation to reduce the pathogen load in the wound as much as possible. In clinical practice, an implant is never placed in an infected wound but in an environment with as little pathogens as possible. However, a contamination of the implants’ surface with a few pathogens from whichever source can always occur, and as little as 100 bacteria are enough to induce a periprosthetic infection [25]. An antimicrobial coating, which is surface active and capable to kill the bacteria trying to adhere to the implant surface adds an additional safety characteristic to the implant while avoiding the potential negative effects of high dose drug release. The remaining planktonic pathogens in the soft tissue can be attacked by the host immune system.

To address the aspects mentioned above, we deviated from established animal models and adapted ours to enable the testing of surface-active coatings without typical release properties and drug efficacy of antibiotics.

The SML coating tested in this study showed a distinct activity against MSSE with a statistically significant reduction of pathogens on the implants surface. Due to the missing or mild infection signs in the clinical observation and histology as well as no systemic effects, the model was considered to be mild, with only a minimal to mild impact on the animal health. The decrease in CFU count of both blank and SML item shows that the immune system of the animals was able to fight the pathogen concentration of ~2 × 10^4^ CFU of MSSE. When compared with the initial pathogen dose, a statistically significant CFU reduction of over 3 Log after explantation on the surface of SML coated items and an over 2 Log reduction in the corresponding rinsing solution and bone marrow was detected. This also significant CFU reduction on the AS^®^ coated blank items was unexpected. Recent papers report similar findings, yet to date, there is no explanation for this effect. This will need to be evaluated further [65].

Comparing the CFU count after explantation, the SML coated items showed a 1.6 Log reduction on both the K-wire surface and the rinsing solution compared to the blank items. In the SML group, 7 of 12 test items were completely free of pathogens, which equals to a cure rate of 58% (*p* = 0.002). On the contrary, only 1 of 12 blank items were free of pathogens, which equals a cure rate of 8% ((*p* = 0.110). These results clearly prove the antimicrobial activity of the SML coating. In addition, the results from the main study are backed up by the results from the two pilot studies, which also showed a clear reduction of pathogens. As the difference in CFU count of blank and SML items was far less pronounced in the bone marrow, this indicates the localized antimicrobial effect of the SML coating.

As no reference values exists that indicate how high the in vivo CFU reduction of an antimicrobial system has to be to prove a certain and clinically relevant antimicrobial activity, it might be hard for the reader to interpret the measured 1.6 Log CFU reduction. To put this into perspective, the clinical reality is to be considered in this context. Each K-wire was implanted with a load of ~20,000 CFU on the K-wire surface. This was done to induce an infection in the animals and subsequently detect a difference in CFU count between SML and blank items. Lower contamination doses were quickly eradicated by the animal’s immune system, and therefore, no effect of the antimicrobial coating could be detected. A contamination of over 20,000 CFU per K-wire equals over 3500 CFUs per cm^2^, which is several orders of magnitud higher than the contamination of a hospital toilet door handle (7.97 ± 0.68 CFU/cm^2^) or a hospital washroom floor (20 CFU/cm^2^) [66,67]. In an orthopedic setting with adequate hygiene management, such high pathogen loads will enter the wound neither through the orthopedic implant, which is delivered sterile, nor through the patient’s skin, which is disinfected and draped before the operation.

At explantation, the mean pathogen count on the surface of a blank K-wire was 9.282 ± 10.585 CFU, and only one of twelve K-wires was pathogen free. On the contrary, on SML coated items, the mean pathogen count was 353 ± 529 CFU, and seven of twelve K-wires were pathogen free, which shows a decrease in pathogen count by the SML coating of roughly 9000 CFU. This is over 3 times the pathogen count that is present at the surface of an PE liner, which is explanted due to PJI (2768 CFUs) [45]. Therefore, the 1.6 Log reduction of pathogens when SML is used is considered to be a substantial safety benefit.

To our knowledge, this is the first established mild osteomyelitis model that works with pre-incubated implants. In this model, we successfully proved an antimicrobial activity of the SML coating.

To date, various orthopedic implants with silver-based surface coatings are approved and used successfully in clinical practice. Within the European Union, to date, these systems are limited to large revision and tumor prosthesis and not used on primary or standard revision implants for total joint arthroplasty. However, as they can only be applied to metal, the PE liner, which is most prone to infection, remains unprotected. A technology that can be applied to both metal and polymer surfaces and that has been proven to reduce adherent bacterial load broadens the possibilities to fight PJI. The SML coating is not designed to be wear resistant, nor has it been tested for wear under continuous loads in this study. In areas with high friction like the joint articulation, the SML coating could be sheared off. It is therefore recommended that for orthopedic implants, the majority of the surfaces be coated to protect against bacterial colonization, but that areas exposed to high wear remain uncoated. Further studies should be conducted to address the osseointegration and wear behavior of the SML coating.

## 5. Conclusions

There is a great need for new infection prophylaxis systems that can improve the safety of patients undergoing joint replacement surgery. We have successfully (i) established a mild osteomyelitis model in rabbits with pre-incubated implants and (ii) demonstrated excellent antimicrobial activity of the presented SML coating. The performed in vitro and in vivo experiments both showed a statistically significant CFU reduction in a clinically relevant scale. The local and systemic silver release remained close to detection limits. Its broad applicability renders the SML coating a promising candidate as an infection prophylaxis system for orthopedic applications.

## Figures and Tables

**Table 1:** Main study-group setup.

Group	Implant	Contamination	Animals	Time of Implantation Post Operation
1	Test item (SML item) K-wire (Titanium/AS^®^) + SML	2 × 10^4^ CFU MSSE RKI 10-00621	12	7 days
2	Blank item K-wire (Titanium/AS^®^)	2 × 10^4^ CFU MSSE RKI 10-00621	12	7 days
3	Negative control: Test item (SML item) K-wire (Titanium/AS^®^) + SML	Without contamination	3	7 days

**Table 2:** Results from the Mann–Whitney test of Log (Implantation) compared to Log (Explantation).

Type	Response	Mean	Difference from Log (Implantation)	*p*-Value Mann–Whitney Test
blank	Log(Implantation)	4.376		
	Log(K-wire)	2.670	−1.706	0.007
	Log(rinsing sol.)	3.393	−0.983	0.069
	Log(BMstd)	2.649	−1.727	0.112
SML	Log(Implantation)	4.336		
	Log(K-wire)	1.073	−3.263	0.000
	Log(rinsing sol.)	1.797	−2.539	0.000
	Log(BMstd)	2.148	−2.188	0.001

Sol. is solution and BMstd is bone marrow standardized.

**Table 3:** Results from the Mann–Whitney test of Blank and SML items after explantation.

Type	Difference	*p*-Value Mann–Whitney Test
Log(K-Wire) Blank vs. SML	1.597	0.022
Log(rinsing sol.) Blank vs. SML	1.596	0.012
Log(BMstd) Blank vs. SML	0.501	0.362

Sol. is solution and BMstd is bone marrow standardized.

## Supplementary Materials

The following are available online at https://www.mdpi.com/1996-1944/13/6/1415/s1, Figure S1: Schematics of band saw cutting of un-demineralized specimens for block preparation. From each of the 5 blocks depicted, 2 section levels spaced at least 50 µm were cut.
