# Supplementary material for: Antimicrobial Silver Multilayer Coating for Prevention of Bacterial Colonization of Orthopedic Implants
Source: Materials (Basel). 2020 Mar 20;13(6):1415. doi: 10.3390/ma13061415 (PMC7143109; doi:10.3390/ma13061415)
Supplement: Supplementary file 1 [file materials-13-01415-s001.pdf]

materials

Supplementary Materials

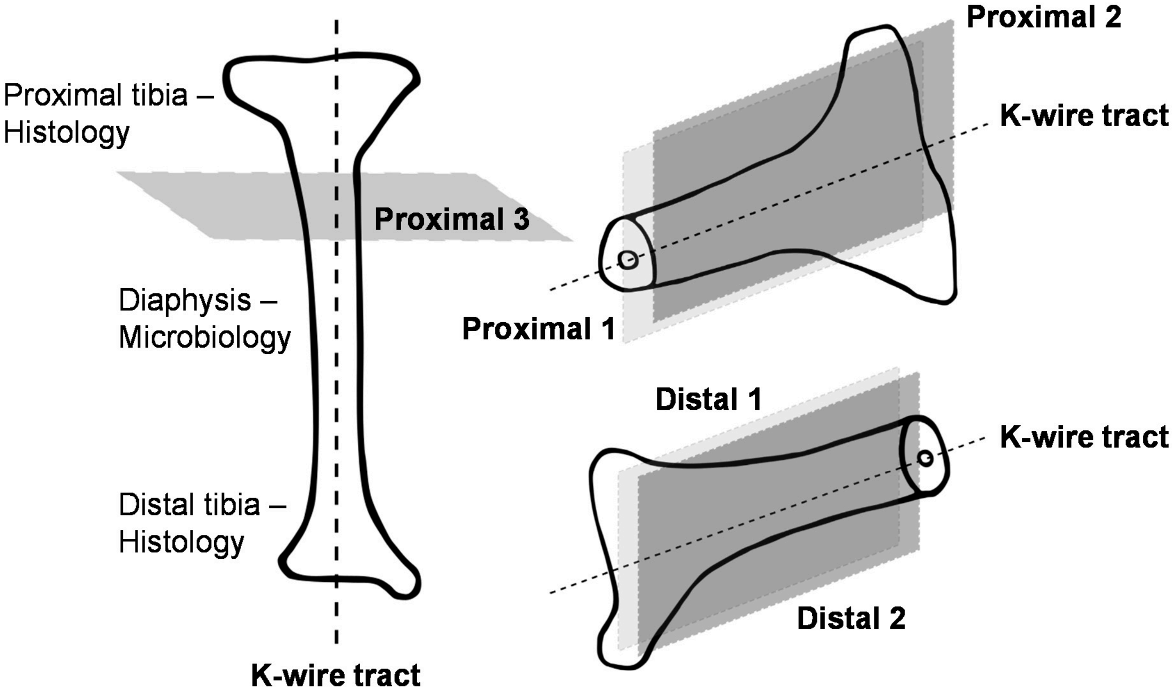

Figure S1: Schematics of band saw cutting of un-demineralized specimens for block preparation. From each of the 5 blocks depicted, 2 section levels spaced at least 50 μm were cut.
